# The Incidence and Recurrence of Getting Lost in Community-Dwelling People with Alzheimer’s Disease: A Two and a Half-Year Follow-Up

**DOI:** 10.1371/journal.pone.0155480

**Published:** 2016-05-16

**Authors:** Ming-Chyi Pai, Chih-Chien Lee

**Affiliations:** 1 Division of Behavioral Neurology, Department of Neurology, National Cheng Kung University Hospital, College of Medicine, National Cheng Kung University, Tainan, Taiwan; 2 Alzheimer’s Disease Research Center, National Cheng Kung University Hospital, Tainan, Taiwan; 3 Institute of Behavioral Medicine, College of Medicine, National Cheng Kung University, Tainan, Taiwan; Eberhard Karls University of Tuebingen Medical School, GERMANY

## Abstract

Getting lost (GL) is a serious problem for people living with Alzheimer’s disease (PwAD), causing psychological distress in both PwAD and caregivers, and increasing the odds of being institutionalized. It is thus important to identify risk factors for the GL events in PwAD. Between April 2009 and March 2012, we invited 185 community-dwelling PwAD and their caregivers to participate in this study. At the baseline, 95 had experienced GL (Group B); the remaining 90 (Group A) had not. We focused on the incidence of GL events and the associated factors by way of demographic data, cognitive function assessed by the Cognitive Ability Screening Instrument (CASI), and spatial navigation abilities as assessed by the Questionnaire of Everyday Navigational Ability (QuENA). After a 2.5-year period, the incidence of GL in Group A was 33.3% and the recurrence of GL in Group B was 40%. Multiple logistic regression analysis revealed that the inattention item on the QuENA and orientation item on the CASI had independent effects on the GL incidence, while the absence of a safety range was associated with the risk of GL recurrence. During the 2.5 years, the PwAD with GL incidence deteriorated more in the mental manipulation item on the CASI than those without. We suggest that before the occurrence of GL, the caregivers of PwAD should refer to the results of cognitive assessment and navigation ability evaluation to enhance the orientation and attention of the PwAD. Once GL occurs, the caregivers must set a safety range to prevent GL recurrence, especially for younger people.

## Introduction

Getting lost (GL) is a serious problem for people living with Alzheimer’s disease (PwAD), causing psychological distress in both PwAD and caregivers, increasing the odds of being institutionalized [1], and sometimes resulting even in fatal consequences [2, 3]. The prevalence of GL in PwAD ranges from 30% to 70% across countries [1, 4–7]. These PwAD may get lost in familiar environments, often when performing daily routine activities [2, 6]. Although the phenomenon is well known and has been widely studied, the predictors for GL are still unclear, making it difficult to arrange appropriate coping strategies.

Previous studies found that PwAD or people with amnesic mild cognitive impairment are more compromised in spatial navigational abilities, such as landmark and scene recognition [8], egocentric/ allocentric orientation [9, 10], and directed attention [11]. Some studies have addressed the risk factors of GL events in such persons, but most are cross-sectional. Kwok et al [4], for example, reported that PwAD with GL events ranked lower on the General Degenerative Scale (GDS). One of our previous studies [12] which examined the relationship between behavioral symptoms of topographical disorientation (TD) and GL events in PwAD revealed that those with a GL history had more severe TD symptoms. Bowen et al [6] conducted a 12-month follow-up study to evaluate the incident GL events among PwAD and described the antecedents and consequences of GL. However, they did not focus on the risk factors or long-term predictors of GL. Only a few longitudinal studies of GL predictors in PwADs [1, 13] have been done. The sample size, however, was small and no attempt was made to differentiate associated factors for incidence and recurrence of GL events. Another study focused on wandering behavior, and did not differentiate GL from wandering. Wandering behavior was defined as aimless or non-goal directed locomotion, including excessive ambulation, eloping behavior, and night-time walking [14–16]. Attention and consciousness may be impaired when the PwAD are wandering, contrast with GL events where PwAD are able to keep a specific goal in mind [17].

Incident and recurrent GL events are quite different. For the former, the PwAD encounter their first ever GL, and before the event their caregivers may be totally unaware that their PwAD are prone to GL, thus no prevention strategies are adopted. Because neither caregiver protection nor change in egression behavior act as confounders, we hypothesized that cognitive impairments and TD symptoms can predict GL incidence. On the contrary, the predictors for GL recurrence in PwAD might be different because of intervention from caregivers as well as restrictions. To this end, we carried out a longitudinal study to ascertain whether any demographic information, cognitive functions or spatial navigation impairments observed by caregivers can predict GL incidence or recurrence in a group of PwAD.

## Methods

### Participants

**Fig 1:**
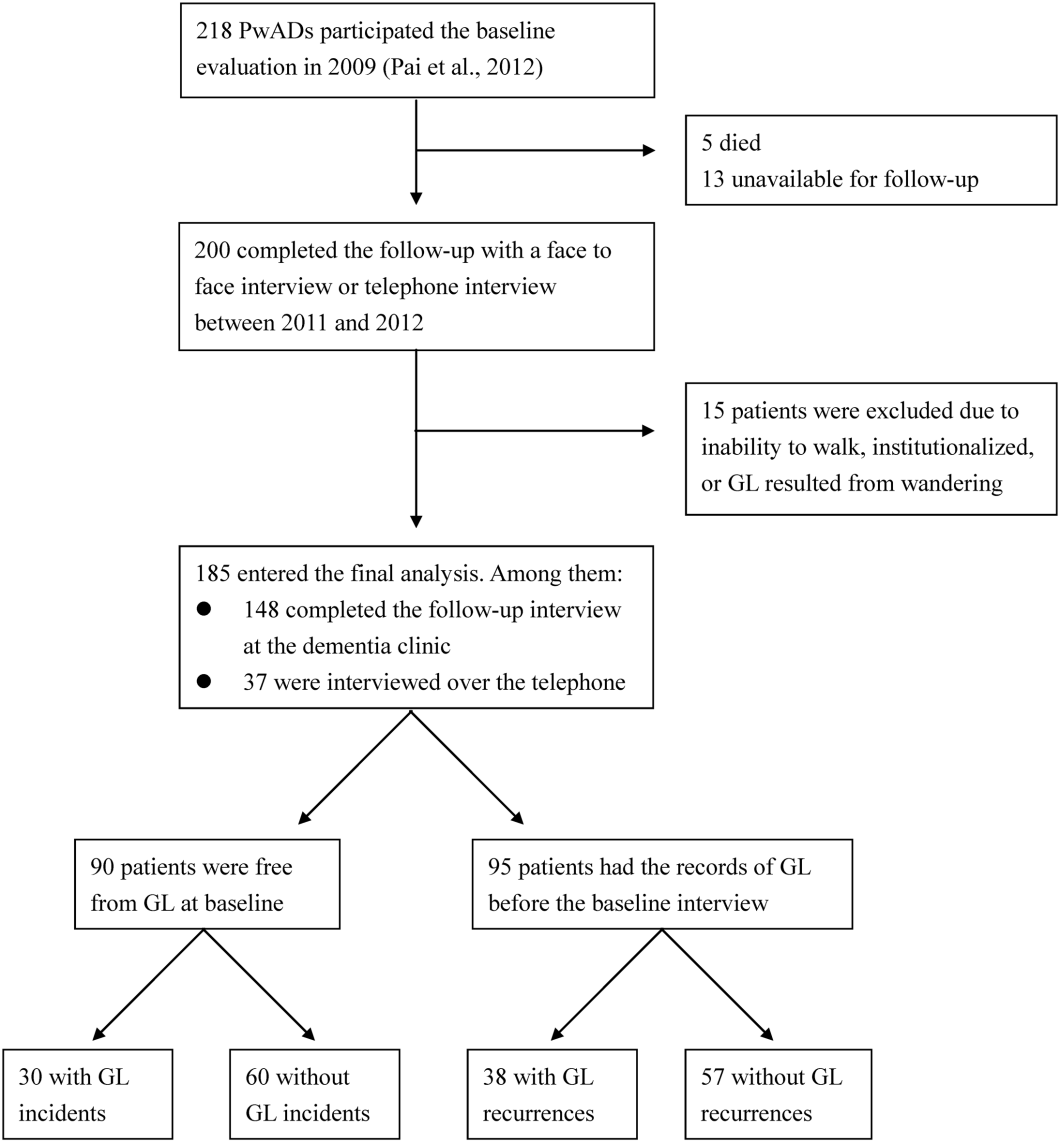
Flow chart of the study participants and Groups. Abbreviation: AD = Alzheimer’s disease; QuENA = Questionnaire of Everyday Navigational Ability; GL = getting lost.

The baseline assessment was carried out from 1 April 2009 to 31 October in 2009, and a follow-up interview was done in the period 1 November 2011 to 31 March 2012 (Fig 1). At baseline, 218 community-dwelling PwADs and their caregivers joined the study [12] for the investigation of new GL events. All the PwAD regularly visited the Alzheimer’s Disease Center of a national university and had no ambulatory problems, aphasia, focal cerebral damage, or visual or auditory impairments. In addition, it was required that they had been living in their current residence before the development of AD. The caregiver had been living with the PwAD and had made adequate observation of their daily life. The PwADs were diagnosed by a senior behavioral neurologist according to the criteria of the National Institute of Neurological and Communicative Disorders and Stroke and the Alzheimer's Disease and Related Disorders Association (NINCDS- ADRDA) and of the *Diagnostic and Statistical Manual of Mental Disorder* (Fourth Edition) and underwent neuropsychological assessment.

During the follow-up period, the PwAD with any of the following conditions were excluded: bed ridden or being restricted by caregivers due to physical handicaps, such as weakness, severe degenerative arthritis, or having been admitted to an institution or nursing home.

### Standard protocol approvals and informed consents

The study was approved by the Institutional Review Board for the Protection of Human Subjects at the National Cheng Kung University Hospital. All subjects gave their written informed consent to participate.

### Definition and assessment of GL

According to the observations of Rowe et al [2], the nature of GL is quite different from that of wandering as noted earlier. In the present study, GL events were assessed by a structural interview and the operational definition of GL was composed as follows: 1) the event occurred in a familiar environment; 2) the PwAD had the sense of “being lost”, or perceived difficulty reaching a goal or returning home; 3) the PwAD were unable to get to their destination without any aid from others; 4) the PwAD had a specific goal or purpose for the excursion and could keep the specific goal in mind along the way; 5) the information was provided by the caregivers because the PwAD may underreport GL events due to memory problems.

### Demographic data and egression behavior

We collected information on age, sex, years of education, disease duration, and factors related to quality and quantity of the excursion, including egression frequency, presence or absence of safety range, and transportation restrictions. The egression frequency was recorded as number of days out per week. People who kept themselves in very familiar surroundings and rarely or never visited less familiar places alone were coded as “with safety range”. PwAD were classified according to transportation restrictions into four levels [18]. The people who could still operate a motorbike, scooter or motor vehicle were gauged minimum restriction (R1) and those who could go out by bicycle were defined as mild restriction (R2). People who went out only on foot were R3. People with the highest restriction (R4) were escorted by caregivers whenever they went out and the escort was usually a spouse, son/ daughter (-in-law), or a full time professional caregiver.

### Global cognitive function

The cognitive function of the PwAD was evaluated by the Cognitive Ability Screening Instrument (CASI) [19] and Mini-Mental State Examination (MMSE) [20] administrated by an experienced clinical psychologist who was blind to the spatial navigation ability and GL records of the PwAD. The CASI includes 9 subscales: remote memory, recent memory, attention, mental manipulation, orientation, abstract thinking, language, visuospatial construction, and verbal fluency. In order to monitor the deteriorative patterns, the changes of the scores between the baseline and follow-up assessment were calculated. CASI is an appropriate tool to distinguish dementia from a cognitively healthy state. Its sensitivity and specificity were 0.80 to 0.90 from previous studies [19, 21, 22].

### Spatial navigational difficulties

The behavioral perspectives of spatial navigation difficulties were evaluated using the Questionnaire of Everyday Navigational Abilities (QuENA) [12], a 10-item scale consisting of 4 factors: landmark and scene agnosia (LSA), egocentric disorientation (ED), heading disorientation (HD), and inattention (INA). Score on the QuENA ranges from 0 to 30, and a higher score reflects more severe TD symptoms. The QuENA has a caregiver’s version (QuENA-C) and patient’s version (QuENA-P). The QuENA-C was used as the main information source reflecting an individual’s true navigation abilities, while the QuENA-P was used to reveal the insight of the PwAD regarding their navigational impairment. The internal consistency, as evaluated by Cronbach’s alpha, is 0.91 and 0.87 for caregiver version and PwAD version respectively, indicating a good internal consistency [12].

### Confounding factors

Many factors not mentioned above may influence the occurrence of new GL events. In order to minimize the possibilities, we collected two types of confounding factors. The first type includes the background variables gathered at the baseline interview, including medication, residential years, self-report maze dull, and caregiver’s educational level. The PwADs were mostly being treated with one of the cholinesterase inhibitors (donepezil, rivastigmine, and galantamine) and some with NMDA antagonist (memantine) or nicergoline, and some both. The self-report maze dull was provided by the PwAD and the caregivers confirmed whether the PwAD had difficulty traveling in new environments before the onset of AD. The educational levels of the caregivers were recorded because they may have an effect on the awareness and the care strategy for their PwAD.

The second type includes changes which might have developed during the follow-up period, including medication changes, moving, failure to follow-up and change in egression frequency. Medication change was defined as any change in generic drugs, including addition, removal, and alteration. We subtracted number of days out per week at follow-up evaluation from baseline data as the change of excursion frequency. Among the PwAD interviewed at baseline, 22.4% were lost to OPD follow-up. We interviewed these PwAD and their caregivers by telephone and evaluated the effect of their medical adherence on GL.

### Statistical analysis

We compared the groups using a t-test with respect to GL incidence and GL recurrence. In order to compare the effect of predictors, we also provided the effect size (Cohen’s d). χ^2^ was used to compare the proportion among groups. Multiple logistic regression analysis was used to estimate the odds ratio (OR) of the GL predictors, and only significant predictors were chosen for further analysis. We conducted three logistic regression analyses separately using a cluster of independent variables: 1) cognitive functions (each subscales of the CASI), 2) spatial navigation difficulty (the severity of TD that derived from the QuENA), 3) self-awareness of spatial navigation difficulty (discrepancy in the QuENA-P and QuENA-C scores and the presence of safety range). Only the significant predictors revealed in the previous analysis qualified for the "composite analysis" to evaluate the magnitude of the predictive power across each cluster. A supplemental analysis was performed to ensure that *the homogeneity* of the interviews conducted face-to-face and by telephone. The analyses were performed using SPSS 15.0.

## Results

As shown in Fig 1, during the follow-up period, among the 218 baseline participants, 5 died and 13 were unreachable. A total of 200 PwAD completed the follow-up evaluation. Among them, 15 were excluded because of weakness and severe degenerative arthritis resulting in inability to ambulate unaided in 7, admission to nursing home in 11, and GL while wandering in 6. Some PwAD met more than one exclusion criteria (inability to ambulate and admission to nursing home in 6, wandering and admission to nursing home in 3). As a result, 185 PwAD entered the final analysis; 148 of these were interviewed face-to-face, and the other 37 by telephone. The average follow-up duration was 2.5 (SD = 0.19) years. The mean age of the 185 participants was 74.9 (SD = 8.6) years, education was 5.9 (SD = 4.9) years, and 121 (65.4%) were female. Because the education level was relatively low in our subjects, the CASI (mean = 60.3, SD = 19.2) and the MMSE (mean = 17.3, SD = 5.6) total score was slightly lower. No difference was detected between the participants interviewed face-to-face and those by telephone across any variables (S1 Table).

### Getting lost events

There were four sub-groups in this study. Group A (without GL events at baseline) and Group B (with GL events at baseline) differed in whether there was a GL event at the baseline. We examined the new GL events during the 2.5 years follow-up duration. In Group A, at follow-up interview, 30 (33.3%) had new GL (INC) and 60 remained free from GL (FFG). In the same way, in Group B, 38 (40%) had new or recurrent GL (REC) and 57 did not (free from recurrent, FFR).

### Predictors of GL incidence in Group A

As shown in Table 1, the demographic data and egression behaviors failed to predict GL incidence, and no difference was detected between Group INC and Group FFG. Regarding global cognitive function, compared with Group FFG, Group INC was worse on baseline MMSE (d = .57, p = .009) and on baseline CASI total score (d = .66, p = .003). Among the CASI subscales, Group INC showed more impairment in remote memory (d = .55, p = .027), orientation (d = .82, p < .001), abstract thinking (d = .61, p = .004), and verbal fluency (d = .56, p = .012) compared with Group FFG. Group INC were worse on the QuENA-C total score (d = .91, p < .001) and on subscales landmark/scene agnosia (d = .78, p < .001), egocentric disorientation (d = .69, p = .002), and inattention (d = .98, p < .001). The discrepancy between the QuENA scores from PwAD and from caregivers were larger in Group INC (d = .63, p = .012). As shown in Table 2, after being adjusted for age, sex, and years of education, logistic regression revealed that GL incidence was predicted by the items of remote memory, orientation, abstract thinking, and verbal fluency on the CASI, and landmark agnosia, egocentric disorientation, inattention, and heading disorientation on the QuENA, and self-awareness of navigation impairments.

**Table 1:** Predictors of getting lost.

	Group A			Group B		
	INC (30)	FFG(60)	*p*1	REC (38)	FFR (57)	*p*2
Age, y, mean ± SD	73.6 ± 9.3	75.9 ± 7.8	.222	71.0 ± 9.3	77.1 ± 7.7	.001
Female, n (%) _a_	17 (56.7)	40 (66.7)	.353	25 (65.8)	39 (68.4)	.788
Years of education, mean ± SD	5.2 ± 5.1	5.9 ± 4.9	.548	6.1 ± 5.2	6.0 ± 4.6	.959
Disease duration, mean ± SD	2.8 ± 2.8	2.7 ± 2.9	.794	2.9 ± 2.6	3.0 ± 2.7	.852
Days out per week, mean ± SD	4.1 ± 2.9	4.4 ± 3.0	.477	3.4 ± 3.0	2.2 ± 3.0	.098
With safety range, n (%) _a_	14 (46.7)	34 (56.7)	.370	22 (57.9)	46 (80.7)	.016
Transport restriction, n(%) _a_			.609			.039
R4, n (%)	6 (20.0)	12 (20.0)		11 (28.9)	32 (56.1)	
R3, n (%)	10 (33.3)	27 (45.0)		10 (26.3)	13 (22.8)	
R2, n (%)	4 (13.3)	4 (6.7)		6 (15.8)	3 (5.3)	
R1, n (%)	10 (33.3)	17 (28.3)		11 (28.9)	9 (15.8)	
MMSE, mean ± SD	15.0 ± 5.8	18.0 ± 4.7	.009	18.5 ± 5.7	17.0 ± 6.0	.242
(range)	(6–28)	(4–28)		(6–25)	(6–27)	
CASI, mean ± SD	51.9 ± 20.5	63.9 ± 15.6	.003	63.3 ± 20.3	59.0 ± 20.1	.310
(range)	(12–89)	(15–91)		(10–92)	(12–92)	
CASI sub-scales						
Remote memory, mean ± SD	7.7 ± 2.5	8.9 ± 1.8	.027	8.5 ± 2.1	8.1 ± 2.3	.396
Recent memory, mean ± SD	3.1 ± 3.3	4.3 ± 2.8	.071	5.0 ± 3.7	4.0 ± 3.4	.177
Attention, mean ± SD	6.4 ± 1.8	6.6 ± 1.1	.474	6.5 ± 1.6	6.6 ± 1.5	.686
Mental manipulation, mean ± SD	5.1 ± 3.5	6.0 ± 3.2	.215	5.6 ± 3.4	5.3 ± 3.1	.718
Orientation, mean ± SD	7.1 ± 5.2	11.2 ± 4.8	.000	10.9 ± 4.8	9.8 ± 6.0	.350
Abstract thinking, mean ± SD	4.8 ± 2.1	6.0 ± 1.8	.004	5.9 ± 1.9	5.5 ± 2.1	.394
Language, mean ± SD	7.2 ± 2.9	8.0 ± 1.3	.131	8.2 ± 1.8	7.6 ± 2.1	.157
Drawing, mean ± SD	6.7 ± 3.3	7.6 ± 2.9	.199	7.5 ± 3.0	7.6 ± 2.8	.794
Verbal fluency, mean ± SD	3.8 ± 2.5	5.1 ± 2.1	.012	5.3 ± 2.8	4.4 ± 2.3	.098
QuENA-C, mean ± SD	8.0 ± 5.6	3.4 ± 4.4	.000	11.4 ± 7.1	11.5 ± 7.8	.969
LSA, mean ± SD	2.7 ± 2.2	1.2 ± 1.6	.000	3.2 ± 2.4	3.6 ± 2.9	.473
ED, mean ± SD	1.7 ± 1.6	0.7 ± 1.3	.002	2.8 ± 1.8	2.6 ± 2.0	.659
INA, mean ± SD	1.8 ± 1.9	0.4 ± 0.7	.000	2.3 ± 2.0	2.3 ± 2.0	.967
HD, mean ± SD	1.7 ± 1.6	1.1 ± 1.7	.085	3.1 ± 2.6	2.9 ± 2.7	.745
Discrepancy score ± SD	5.3 ± 6.5	1.9 ± 4.1	.012	5.0 ± 7.1	6.1 ± 7.9	.486

**Abbreviations**: Group A = without any GL records at baseline; Group B = with one or more GL events before baseline; INC = with GL incidence; FFG = remaining free from GL; REC = with GL recurrence; FFR = free from GL recurrence; R4 = escorted by caregivers when going out; R3 = can only go out alone on foot; R2 = can still cycle a bike around the area; R1 = can still operate a motorbike or car; QuENA-C = Questionnaire of Everyday Navigational Ability, caregiver version; LSA = landmark and scene agnosia; ED = egocentric disorientation; INA = inattention; HD = heading disorientation; *p*1 = *p* value within Group A; *p*2 = *p* value within Group B.

^a^ Analyzed by Pearson’s Chi-square and the percentage was within GL events.

**Table 2:** The predictive power of variables of interest for new GL ocurrence*.

Risk factors for GL	GL incidence			GL recurrence		
	OR	95% CI	*p*1	OR	95% CI	*p*2
Demographic data						
Age (per year decrement)	1.04	.983~1.09	.184	1.09	1.03~1.13	.002
Male vs. female	1.95	.715~5.31	.192	1.42	.501~4.01	.511
Years of education (per year increment)	.940	.850~1.04	.235	.979	.883~1.09	.694
MMSE (per point increment) _a_	.862	.770~.965	.010	1.01	.926~1.10	.812
CASI subscales (per point increment) _a_,						
Remote memory	.732	.576~.930	.011	.996	.795~1.25	.971
Recent memory	.838	.707~1.02	.062	1.03	.900~1.18	.676
Attention	.883	.635~1.23	.462	.845	.623~1.15	.280
Mental manipulation	.881	.743~1.05	.147	.960	.819~1.13	.612
Orientation	.824	.733~.926	.001	1.00	.920~1.10	.934
Abstract thinking	.651	.486~.873	.004	.997	.781~1.27	.983
Language	.795	.628~1.06	.066	1.04	.810~1.33	.754
Visual construction	.907	.771~1.07	.237	.954	.804~1.13	.590
Verbal fluency	.735	.588~.919	.007	1.05	.866~1.28	.609
QuENA-C (per point increment) _a_						
Landmark and scene agnosia	1.62	1.21~2.17	.001	.983	.826~1.17	.852
Egocentric disorientation	1.68	1.19~2.37	.003	1.13	.892~1.43	.312
Inattention	2.46	1.55~3.90	.000	1.02	.812~1.27	.891
Heading disorientation	1.36	1.01~1.81	.040	1.04	.880~1.23	.636
Self-awareness _a_						
Discrepancy score (per point increment)	1.15	1.05~1.27	.004	.981	.921~1.04	.543
No safety range vs. with safety range	1.23	.447~3.41	.685	3.36	1.07~10.53	.037
Egression behavior _a_						
Days out per week (per day increment)	.920	.784~1.08	.307	1.18	.960~1.30	.152
Restriction of transport vs. R4						
R3	.405	.117~1.40	.154	2.29	.743~7.05	.194
R2	.640	.121~3.40	.600	3.85	.843~20.14	.062
R1	.390	.101~1.50	.172	1.93	.470~5.34	.306

Abbreviations: GL = getting lost; OR = odds ratio; QuENA = Questionnaire of Everyday Navigational Ability; R4 = escorted by caregivers when going out; R3 = can only go out alone on foot; R2 = can still cycle a bike around the area; R1 = can still operate a motorbike or car; *p*1 = *p* value within GL incidence; *p*2 = *p* value within GL recurrence.

^a^ Adjusted for age, sex, and years of education.

*The predictors were entered into each model seperately.

### Predictors of GL recurrence (Group B)

Regarding GL recurrence, the predictive variables in Group B were quite different from those in Group A. As shown in Tables 1 and 2, Group REC was younger (d = .71, p = .001) which carried a higher risk to develop recurrent GL. The absence of a safety range increased the risk of GL recurrence markedly as well. Although the transportation restriction had an effect on recurrence, the predictive power was diminished when age, sex, and education were adjusted. Neither general cognitive function nor the navigation impairments can predict GL recurrence.

### The independent effect of predictors

To evaluate the magnitude of the power of predictors, we put the significant variables in the previous analysis into a model simultaneously and adjusted for age, sex, and education. As shown in Table 3, the GL incidence can be predicted by orientation subscale in the cognitive cluster (CASI), inattention in the behavioral cluster (QuENA), and the discrepancy score in self-awareness, while only the development of a safety range can predict GL recurrence. Again, we put those predictors into the composite analysis and found that the effect of the discrepancy score in self-awareness was diminished, and the orientation and inattention still had an effect on GL incidence. Meanwhile, the predictive power of the safety range remained robust in the final model regarding GL recurrence.

**Table 3:** Independent effects of predictors for new GL occurrence*.

Risk factors for GL	GL incidence			GL recurrence		
	OR	95% CI	*p*1	OR	95% CI	*p*2
Cognitive function (per point increment) _a_						
MMSE	1.17	.920~1.50	.196	1.01	.833~1.23	.908
Remote memory	1.03	.700~1.51	.895	.968	.708~1.33	.836
Orientation	.790	.650~.961	.018	.990	.851~1.15	.895
Abstract thinking	.746	.482~1.15	.187	.952	.668~1.36	.788
Verbal fluency	.817	.614~1.09	.163	1.09	.809~1.46	.580
Behavioral manifestation (per point increment) _a_						
Landmark and scene agnosia	1.41	.788~1.02	.159	.873	.680~1.12	.288
Egocentric disorientation	1.50	.860~2.61	.153	1.29	.894~1.86	.147
Inattention	2.13	1.28~3.56	.004	.964	.731~1.27	.793
Heading disorientation	.700	.416~1.18	.181	1.01	.793~1.29	.937
Self-awareness _a_						
No safety range vs. with safety range	.867	.297~2.53	.749	3.80	1.14~12.66	.029
Discrepancy score (per point increment)	1.13	1.02~1.24	.015	.972	.909~1.04	.401
Composite analysis _a__,_ _b_						
Orientation	.830	.714~.964	.015	.974	.875~1.08	.633
Inattention	1.85	1.08~3.16	.026	.983	.740~1.31	.907
No safety range	1.03	.313~3.39	.960	4.26	1.27~14.29	.019
Discrepancy score	1.06	.928~1.22	.379	.969	.899~1.04	.402

Abbreviations: GL = getting lost; OR = odds ratio; *p*1 = p value within Group A; *p*2 = p value within Group B.

^a^ Adjusted for age, sex, and years of education.

^b^ Including only previous significant measures.

*Values were determined by putting the predictors into each model (or cluster) simultaneously.

### Deteriorative patterns

The mental deterioration was derived by subtracting follow-up scores from baseline scores. 11 were excluded from the analysis because of no second neuropsychological assessment. As shown in Table 4, Group FFR and Group REC deteriorated on the CASI and MMSE scores equally. Similar findings were observed between INC and FFG, while INC deteriorated more in mental manipulation than FFG did (d = .57, p = .031).

**Table 4:** Deterioration in neuropsychological test*.

	Group A			Group B		
	INC (27)	FFG (59)	*p*1	REC (36)	FFR (52)	*p*2
MMSE, mean ± SD	1.67 ± 4.6	1.1 ± 4.0	.575	.50 ± 4.6	.84 ± 3.2	.681
CASI, mean ± SD	8.3 ± 16.9	5.3 ± 11.2	.403	3.8 ± 14.9	4.6 ± 9.8	.745
CASI sub-scales						
Remote memory, mean ± SD	.56 ± 2.1	.81 ± 2.2	.590	.42 ± 2.7	.31 ± 1.6	.810
Recent memory, mean ± SD	.89 ± 1.8	.18 ± 2.2	.147	.16± 2.8	-.34 ± 2.0	.320
Attention, mean ± SD	.56 ± 1.7	.34 ± 1.3	.521	.53 ± 1.4	.13 ± .97	.124
Mental manipulation, mean ± SD	1.6 ± 2.9	.15 ± 2.1	.031	.58 ± 3.3	.87 ± 2.5	.648
Orientation, mean ± SD	1.4 ± 4.5	1.5 ± 4.8	.941	.33 ± 3.9	1.1 ± 3.0	.283
Abstract thinking, mean ± SD	.70 ± 2.1	.46 ± 1.6	.555	.06 ± 1.6	.27 ± 1.3	.493
Language, mean ± SD	.62 ± 2.2	.22 ± 1.6	.339	.61 ± 2.1	.25 ± 1.9	.401
Drawing, mean ± SD	1.1 ± 3.6	.86 ± 2.7	.651	.69 ± 2.1	1.3 ± 2.6	.226
Verbal fluency, mean ± SD	.77 ± 2.6	-.18 ± 8.1	.549	.67 ± 1.8	.67 ± 2.1	.988

**Abbreviations**: Group A = without any GL records at baseline; Group B = with one or more GL events before baseline; INC = with GL incidence; FFG = remaining free from GL; REC = with GL recurrence; FFR = free from GL recurrence; *p*1 = *p* value within Group A; *p*2 = *p* value within Group B.

*The change in scores was derived by subtracting follow-up scores from baseline scores.

### Confounding factors

Of the PwAD recruited in 2009, 11 had been admitted to nursing homes and were excluded from our final analysis. One who shifted residency among his sons was excluded according to the criteria (GL out of wandering). All of the PwAD entering the final analysis had not moved during the follow-up period. No effect of the confounding factors on either GL incidence or recurrence was detected and none of the factors can predict GL incidence and recurrence (see S2–S4 Tables).

## Discussion

### Incidence and recurrence

Previous studies have demonstrated that spatial navigation impairments in PwADs occur not simply in learning new environments [23–25] but also in tasks using familiar materials. [8, 26] Consequently, PwAD may get lost unexpectedly in familiar surroundings on a routine journey [2, 6]. In fact, GL is among the incipient symptoms in some PwAD, and most of the them had their first GL experience within two years of the clinical onset of AD [5, 17]. Our findings are consistent with these reports. Strikingly, at the 2.5-year follow-up interview, about two-thirds of the participants had had GL events in familiar surroundings. These PwAD usually remained competent in daily excursions and their GL events were not due to wandering.

### The predictive values of factors

#### Incidence

The performance of subscale orientation on the baseline CASI and the presence of inattention on the QuENA are contributory to the risk of GL incidence in Group A. To complete the CASI orientation subscale successfully, participants need multiple cognitive abilities to update the information of their current situation including a consistent and reliable integration of attention, perception, and memory [27]. In PwAD, the impairments of orientation correlate well with hypometabolism in the posterior cingulate cortex (PCC) [28–30] which is known to play an important role in memory-related processes, including recognition of familiar places [31, 32] and retrieval of autobiographical memory [33]. In our previous cross-sectional study of a similar population, no difference in cognitive function was detected between PwADs who had experienced GL and those who had not [12]. Only a longitudinal study like the present one can reveal the effect of specific cognitive functions on the GL incidence. For example, compared with Group FFG, Group INC was worse in cognitive functions including remote memory, orientation, abstract thinking, verbal fluency, but not in recent memory. In addition, the score of mental manipulation of Group INC dropped markedly over the follow-up period compared with that of Group FFG, see Table 4. In spite of the complicated mechanisms contributing to spatial navigation impairment in PwAD, the role of specific cognitive functions was demonstrated in this study. Meanwhile, the inattention addressed in the QuENA reflects the careless mistakes PwAD may make during daily navigation, in particular when they are in less familiar or novel environments. When inattention and disorientation happens to a PwAD, he or she may fail to update and integrate the ongoing navigational events. Consequently, the individual may encounter difficulty getting back on the correct path and a GL outcome may ensue. Located in southern Taiwan, Tainan was the capital of Taiwan 350 years ago. Although Tainan has been modernized over the past decades, the streets and buildings in downtown Tainan are still highly complex and dense. Therefore, for successful navigation in downtown Tainan extensive decision making is required to negotiate the numerous intersections, which is a challenge to those PwAD with impaired executive functions [34] and the risk of GL rises.

#### Recurrence

As mentioned earlier, Group A and Group B differed in whether there was a GL event at the baseline. Before the first ever GL event, the PwAD and caregivers probably had no awareness of, or underestimated the risk of GL. Once GL occurred, however, not all PwAD and their caregivers would take action to prevent GL recurrence [17].

The factors relevant to GL recurrence are quite different from those relevant to GL incidence. The neuropsychological and behavioral variables failed to predict any GL recurrence in PwAD. On the contrary, a younger age was the most reliable predictor of GL recurrence while the development of a safety range was a protector. A safety range indicates that the PwAD restricts himself/ herself to a very familiar territory and refuses to go to less familiar places. However, the reasons leading to the establishment of a safety range remain unknown in the present study. In the process of interviewing, some PwAD claimed that the safety range resulted from "external" reasons, such as older age, poor physical condition, fear of traffic accidents, or restrictions imposed by the caregiver. Future studies might focus on the reasons leading to the establishment of a safety range. On the other hand, the insight of TD symptoms (the discrepancy score between QuENA-P and QuENA-C) which played an important role in the prevention of GL incidence was an "internal" cue serving to warn PwAD against GL. In other words, for those PwAD without GL experience, raising awareness of TD may decrease the risk of GL, whereas for those with a history of GL, the establishment of a safety range may be more effective in preventing GL recurrence.

As for the restrictions set by the caregivers, most caregivers acknowledged the GL risk and would do their best to accompany their PwAD in outdoor daily activities. In the present study, however, even when the PwAD were escorted by caregivers outside (R4), the risk of new GL occurrence was not entirely eliminated. Some PwAD had a strong desire to go out alone, especially the younger ones, while the caregivers could not provide 24-hour monitoring. Moreover, the caregivers might consider the PwAD’s familiar environments safe, but in fact GL events can still occur even in familiar environments as AD progresses [8, 16]. As a result, the caregivers should examine the wayfinding behavior of the PwAD even in their familiar territories. When a person with AD experiences one or more GL events and his/ her age is relatively young, the caregivers should provide some GL coping strategies for their PwAD, such as the use of GPS devices, to minimize the resulting inconvenience.

Interestingly, about one-third of the participants remained free of GL 5 years after their clinical onset. These participants, or Group FFG, performed better in global cognitive functions compared with those already having experienced GL within the 2.5-year follow-up. They also showed a better spatial navigation abilities (p < 0.001, df = 3) and better self-appraisal of TD symptoms (p = 0.005, df = 3) than the other groups. Likewise, Group B had their first GL event earlier than Group A in the clinical course of AD which indicates that GL was among the incipient symptoms for Group B but not for Group A. Whether these differences resulted from the progression of AD, PwAD's premorbid ability, or the effect of the subtype variation of clinical AD [35, 36] is unknown. Previous studies proposed the existence of subtypes under the diagnosis of AD according to the heterogeneity of neuropathology or clinical manifestation [35, 37, 38]. Although the number of subtypes may differ from one study to another because of methodological issues, the primary classifications are typical and atypical AD; the former is characterized by anterograde episodic memory impairment and the latter may include other cognitive deficits such as impairment in abstract reasoning, and verbal fluency [39]. The neuropathology of typical AD is manifested in greater damage to the hippocampi and related structures, while the atypical type shows is more damaged to the extra-hippocampal areas such as the inferior parietal cortex, middle frontal cortex, posterior cingulate cortex, and retrosplenial cortex [37, 40, 41]. The retrosplenial cortex, in particular, is related to heading orientation or the translation between different spatial representations which is very important in spatial navigation [42].

This study is of value due to its longitudinal observation which can help identify predictors of GL incidence and recurrence in PwAD. Even so, this study has some limitations. The cognitive tests used in the study, such as the Route Map Recall Test published elsewhere [43], were not specific to navigation ability. However, since the global cognitive test can be routinely administrated, it might be of help for early detection of GL risk. Secondly, some of the informants were not the same at baseline and follow-up interviews. Nevertheless, the new informants at the follow-up interview usually confirmed the history of previous GL occurrence of their PwAD given by the prior informants at the baseline. Finally, some GL events may be ignored by caregivers resulting in an underestimate of the incidence or recurrence of GL events. Moreover, we took only one assessment at the follow-up period which may raise issues of reliability. Thus the reporting of GL events should be interpreted coutiously. Future studies may use wearable devices such as GPS rings to record the actual GL events and give a more precise description of GL behavior, such as the distance away from familiar routes or details of ineffective wayfinding attempts.

In summary, GL incidence in PwADs can be predicted by neuropsychological and behavioral factors while the development of a safety range can help prevent GL recurrence. Caregivers are strongly urged to provide structured and friendly environments to enhance the orientation and attention of their PwAD, to remind them of the dangers of going out alone, and to evaluate their navigation ability carefully.

This paper was presented at Alzheimer's Association International Conference on July 13–18, 2013 in Boston, United States. No company provided support of any kind for this study. Dr. Pai reports having served as a consultant and/or having received lecture fees from Janssen, Lilly, Novartis, and Eisai. This does not alter our adherence to PLOS ONE policies on sharing data and materials. Mr. Lee has no financial conflicts of interest.

## Supporting Information

S1 Table Variables between face to face interview and telephone interview. Abbreviations: GL = getting lost; QuENA = Questionnaire of Everyday Navigational Ability; LSA = landmark and scene agnosia; ED = egocentric disorientation; INA = inattention; HD = heading disorientation. _a_ Analyzed by Pearson’s Chi-square and the percentages were within GL events.
(DOC)

S2 Table The drug use history and GL risks. Abbreviation: GL = getting lost; *p*1 = p value within Group A; *p*2 = p value within Group B. _a_ Analyzed by Pearson’s Chi-square, and the percentages were within GL events.
(DOC)

S3 Table Confounding factors of GL incidence and recurrence in the PwAD. Abbreviations: GL = getting lost; PwAD = people with Alzheimer's disease; Group A = without any GL records at baseline; Group B = with one or more GL events before baseline; INC = with GL incidence; FFG = remaining free from GL; REC = with GL recurrence; FFR = free from GL recurrence; *p*1 = p value within Group A; *p*2 = p value within Group B. _a_ Analyzed by Pearson’s Chi-square, and the percentages were within GL events. *The changes were derived by subtracting follow-up scores from baseline scores.
(DOC)

S4 Table Other GL related factors _a_. Abbreviations: GL = getting lost; OR = odds ratio; *p*1 = p value within Group A; *p*2 = p value within Group B. _a_ Adjusted for age, sex, and years of education.
(DOC)
