# Supplementary material for: The Incidence and Recurrence of Getting Lost in Community-Dwelling People with Alzheimer’s Disease: A Two and a Half-Year Follow-Up
Source: PLoS One. 2016 May 16;11(5):e0155480. doi: 10.1371/journal.pone.0155480 (PMC4868297; doi:10.1371/journal.pone.0155480)
Supplement: S1 Table — Abbreviations: GL = getting lost; QuENA = Questionnaire of Everyday Navigational Ability; LSA = landmark and scene agnosia; ED = egocentric disorientation; INA = inattention; HD = heading disorientation. a Analyzed by Pearson’s Chi-square and the percentages were within GL events. (DOC) [file pone.0155480.s001.doc]

**S1 Table. Variables between face to face interview and telephone interview**

| Variables | Face to face (148) | Telephone (37) | *p* |
| --- | --- | --- | --- |
| Age, y, mean ± SD | 75.1 ± 8.3 | 74.7 ± 10.9 | .715 |
| Female , n (%) _a_ | 96 (64.9) | 25 (67.6) | .795 |
| Years of education, mean ± SD | 5.8 ± 5.2 | 5.9 ± 4.8 | .937 |
| Residential years, mean ± SD | 25.5 ± 23.3 | 28.8 ± 20.0 | .401 |
| Disease duration, mean ± SD | 2.2 ± 2.6 | 2.9 ± 2.8 | .137 |
| Days out per week, mean ± SD | 3.8 ± 2.8 | 3.5 ± 2.9 | .637 |
| GL incidence (73 vs. 17), n (%) _a_ | 23 (31.5) | 7 (41.2) | .446 |
| GL recurrence (75 vs. 20), n (%) _a_ | 30 (40.0) | 8 (40.0) | 1.00 |
| With safety range, n (%) _a_ | 91 (61.1) | 25 (69.4) | .493 |
| MMSE, mean ± SD | 17.1 ± 3.1 | 16.2 ± 3.1 | .116 |
| CASI, mean ± SD | 60.0 ± 19.2 | 60.3 ± 18.5 | .931 |
| Remote memory, mean ± SD | 8.3 ± 2.3 | 8.4 ± 2.0 | .809 |
| Recent memory, mean ± SD | 4.4 ± 3.2 | 4.3 ± 3.3 | .865 |
| Attention, mean ± SD | 6.5 ± 1.5 | 6.5 ± 1.3 | .998 |
| Mental manipulation, mean ± SD | 5.5 ± 3.2 | 5.3 ± 3.1 | .733 |
| Orientation, mean ± SD | 9.8 ± 5.5 | 10.9 ± 5.4 | .276 |
| Abstract thinking, mean ± SD | 5.6 ± 2.0 | 5.4 ± 2.0 | .589 |
| Language, mean ± SD | 7.7 ± 2.1 | 7.7 ± 2.1 | .989 |
| Drawing, mean ± SD | 7.5 ± 3.0 | 6.8 ± 3.1 | .209 |
| Verbal fluency, mean ± SD | 4.6 ± 2.4 | 4.8 ± 2.3 | .645 |
| QuENA, mean ± SD | 8.4 ± 7.6 | 7.8 ± 6.4 | .658 |
| LSA, mean ± SD | 2.7 ± 2.6 | 2.4 ± 2.3 | .523 |
| ED, mean ± SD | 1.9 ± 1.9 | 1.7 ± 1.4 | .548 |
| INA, mean ± SD | 1.6 ± 1.9 | 1.6 ± 1.9 | .968 |
| HD, mean ± SD | 2.3 ± 2.5 | 2.1 ± 2.6 | .667 |
| Discrepancy score, mean ± SD | 4.7 ± 6.8 | 3.1 ± 6.0 | .192 |

Abbreviations: GL = getting lost; QuENA = Questionnaire of Everyday Navigational Ability; LSA = landmark and scene agnosia; ED = egocentric disorientation; INA = inattention; HD = heading disorientation.

a Analyzed by Pearson’s Chi-square and the percentages were within GL events.
