# Supplementary material for: The Incidence and Recurrence of Getting Lost in Community-Dwelling People with Alzheimer’s Disease: A Two and a Half-Year Follow-Up
Source: PLoS One. 2016 May 16;11(5):e0155480. doi: 10.1371/journal.pone.0155480 (PMC4868297; doi:10.1371/journal.pone.0155480)
Supplement: S2 Table — Abbreviation: GL = getting lost; p1 = p value within Group A; p2 = p value within Group B. a Analyzed by Pearson’s Chi-square, and the percentages were within GL events. (DOC) [file pone.0155480.s002.doc]

**S2 Table. The drug use history and GL risks**

| Risk factors for GL | Group A | | | Group B | | |
| --- | --- | --- | --- | --- | --- | --- |
|  | INC (30) | FFG (60) | *p*1 | REC (38) | FFR (57) | *p*2 |
| Drug use history in 2009 _a_ |  |  | .184 |  |  | .601 |
| Donepezil (%) | 11 (36.7) | 35 (58.3) |  | 15 (39.5) | 23 (40.4) |  |
| Rivastigmine (%) | 6 (20.0) | 12 (20.0) |  | 6 (15.8) | 10 (17.5) |  |
| Galantamine (%) | 5 (16.7) | 7 (11.7) |  | 10 (26.3) | 12 (21.1) |  |
| Memantine (%) | 4 (13.3) | 2 (3.3) |  | 6 (15.8) | 6 (10.5) |  |
| Nicergoline (%) | 4 (13.3) | 4 (6.7) |  | 1 (2.6) | 6 (10.5) |  |

Abbreviation: GL = getting lost; Group A = without any GL records at baseline; Group B = with one or more GL events before baseline; INC = with GL incidence; FFG = remaining free from GL; REC = with GL recurrence; FFR = free from GL recurrence; *p*1 = the *p* value within Group A; *p*2 = the *p* value within Group B.

a Analyzed by Pearson’s Chi-square, and the percentages were within GL events.
