# Supplementary material for: The Incidence and Recurrence of Getting Lost in Community-Dwelling People with Alzheimer’s Disease: A Two and a Half-Year Follow-Up
Source: PLoS One. 2016 May 16;11(5):e0155480. doi: 10.1371/journal.pone.0155480 (PMC4868297; doi:10.1371/journal.pone.0155480)
Supplement: S3 Table — Abbreviations: GL = getting lost; PwAD = people with Alzheimer's disease; Group A = without any GL records at baseline; Group B = with one or more GL events before baseline; INC = with GL incidence; FFG = remaining free from GL; REC = with GL recurrence; FFR = free from GL recurrence; p1 = p value within Group A; p2 = p value within Group B. a Analyzed by Pearson’s Chi-square, and the percentages were within GL events. *The changes were derived by subtracting follow-up scores from baseline scores. (DOC) [file pone.0155480.s003.doc]

**S3 Table. Confounding factors of GL incidence and recurrence in the PwAD**

|  | Group A | | | Group B | | |
| --- | --- | --- | --- | --- | --- | --- |
|  | INC (30) | FFG (60) | *p*1 | REC (38) | FFR (57) | *p*2 |
| Variables at baseline |  |  |  |  |  |  |
| Self-report maze dull, n (%) _a_ | 2 (6.7) | 4 (6.7) | 1.00 | 5 (13.1) | 11 (19.3) | .433 |
| Residential years, mean ± SD | 31.9 ± 25.4 | 26.7 ± 20.3 | .317 | 26.6 ± 18.8 | 28.1 ± 20.1 | .896 |
| Caregiver’s education, mean ± SD | 12.3 ± 4.7 | 12.9 ± 4.9 | .653 | 14.3 ± 4.0 | 12.2 ± 3.4 | .145 |
| Disease duration, mean ± SD | 2.8 ± 2.7 | 2.7 ± 2.9 | .791 | 2.5 ± 2.8 | 2.9 ± 2.7 | .453 |
| Changes within the 2.5-year |  |  |  |  |  |  |
| Drug change, n (%) _a_ | 8 (26.7) | 11 (18.3) | .361 | 10 (23.1) | 13 (22.8) | .696 |
| Loss of follow-up in the clinic, n (%) _a_ | 7 (23.3) | 10 (16.7) | .446 | 8 (21.1) | 12 (21.1) | 1.00 |
| Days out per week (change), mean ± SD* | 1.6 ± 2.4 | 0.9 ± 1.6 | .120 | 2.7 ± 2.7 | 2.3 ± 2.8 | .656 |

Abbreviations: GL = getting lost; PwAD = people with Alzheimer's disease; Group A = without any GL records at baseline; Group B = with one or more GL events before baseline; INC = with GL incidence; FFG = remaining free from GL; REC = with GL recurrence; FFR = free from GL recurrence; *p*1 = p value within Group A; *p*2 = p value within Group B.

a Analyzed by Pearson’s Chi-square, and the percentages were within GL events.

* The changes were derived by subtracting follow-up scores from baseline scores.
