# Supplementary material for: The Incidence and Recurrence of Getting Lost in Community-Dwelling People with Alzheimer’s Disease: A Two and a Half-Year Follow-Up
Source: PLoS One. 2016 May 16;11(5):e0155480. doi: 10.1371/journal.pone.0155480 (PMC4868297; doi:10.1371/journal.pone.0155480)
Supplement: S4 Table — Abbreviations: GL = getting lost; OR = odds ratio; p1 = p value within Group A; p2 = p value within Group B. a Adjusted for age, sex, and years of education. (DOC) [file pone.0155480.s004.doc]

**S4 Table. Other GL related factors _a_**

| Risk factors for GL | GL incidence | | |  | GL recurrence | | |
| --- | --- | --- | --- | --- | --- | --- | --- |
|  | OR | 95% CI | *p*1 |  | OR | 95% CI | *p*2 |
| Residential years | 1.01 | .990~1.03 | .280 |  | .999 | .975~1.02 | .947 |
| Self-report maze dull | .839 | .134~5.25 | .852 |  | .551 | .158~1.91 | .348 |
| Years of education (caregiver) | 1.00 | .886~1.13 | .996 |  | 1.22 | .998~1.46 | .075 |
| Disease duration | 1.05 | .895~1.24 | .536 |  | 1.00 | .851~1.19 | .929 |
| Drug change | 1.85 | .631~5.45 | .262 |  | 1.02 | .358~2.88 | .978 |
| Loss of follow-up in the clinic | 1.93 | .613~6.07 | .262 |  | .674 | .219~2.08 | .492 |
| Days out per week (change) | 1.04 | .991~1.09 | .113 |  | 1.01 | .980~1.04 | .515 |

Abbreviations: GL = getting lost; OR = odds ratio; *p*1 = *p* value within Group A; *p*2 = *p* value within Group B.

a Adjusted for age, sex, and years of education.
